# Morphofunctional Nutritional Assessment in Clinical Practice: A New Approach to Assessing Nutritional Status

**DOI:** 10.3390/nu15194300

**Published:** 2023-10-09

**Authors:** Daniel-Antonio de Luis Roman, Juan José Lopez Gomez

**Affiliations:** 1 Servicio de Endocrinología y Nutrición, Hospital Clínico Universitario de Valladolid, 47003 Valladolid, Spain; 2 Instituto de Endocrinología y Nutrición (IENVA), Universidad de Valladolid, 47003 Valladolid, Spain

This Special Issue of *Nutrients* titled “Morphofunctional Nutritional Assessment in Clinical Practice” is oriented to the diagnosis of disease-related malnutrition (DRM). Disease-related malnutrition is a highly prevalent pathology which has become a great challenge to healthcare systems. This disease has a prevalence between 20 and 50% in hospitalized patients [1,2]. Malnutrition can be associated with other conditions such sarcopenia, defined as a loss of muscle mass and function. This disease was described as a primary condition associated with aging and frailty but the European Working Group on Sarcopenia in Older People (EWGSOP2) highlighted that secondary sarcopenia is associated with several diseases [3]. The adequate diagnosis of malnutrition and sarcopenia are based in tests to evaluate dietary intake, body composition, muscle strength and function, and biochemical parameters, which is called morphofunctional nutritional assessment [4]. However, the diagnosis of DRM is difficult because it does not depend only on body weight at a given time, but also on its evolution and the underlying pathological situations; thus, malnutrition is often underdiagnosed and undertreated [5], and is a serious health risk to patients. Therefore, the clinical use of body composition measurements is essential for the adequate assessment of this malnutrition, especially in the evaluation of muscle mass and function. In this context, nutritional assessment can no longer be based on classical anthropometric measurements. The concept of morphofunctional nutritional assessment postulates that the diagnosis and monitoring of nutritional status must be carried out using techniques and biomarkers that evaluate intake, anthropometry, body composition, muscle strength and function, which include techniques such as bioelectrical impedance analysis or nutritional ultrasound, and new biological parameters as well. This new diagnostic approach can help us to evaluate patients at risk of malnutrition and allow for the early diagnosis of DRM and personalized treatment for this condition.

Therefore, we are faced with a transition period in the area of nutritional assessment and there is no global consensus on the approach to DRM assessment. Many parameters have been used, such as body weight loss, body mass index, muscle mass, or dietary intake, which are included in most malnutrition screening tools [6], while other techniques, such as functional parameters, have gradually gained attention [7]. Nowadays, the criteria of DRM established by the Global Leadership Initiative on Malnutrition (GLIM) has enabled a more comprehensive nutritional assessment by including the evaluation of muscle mass, disease inflammation, and dietary intake [8]. The evaluation of body composition, especially muscle mass, is an important component of the diagnosis of malnutrition and sarcopenia, and it plays an essential role in monitoring the nutritional treatment of DRM. Nevertheless, the diagnosis of muscle quantity and quality is also difficult. Some techniques are not accurate such as anthropometric parameters or that use estimations based on bioimpedanciometry. Moreover, there are some tests like computerized tomography and magnetic resonance imaging that are considered gold standards but are more expensive, with potential side effects and are not feasible in routine clinical practice [9].

In this context, new easy and cheap techniques such as ultrasonography have demonstrated utility in morphofunctional evaluation. For example, parameters of the phase angle of BIA were correlated with muscle area through ultrasound, muscle echo intensity of the rectus femoris of the quadriceps, serum protein, quality of life SF-36, and strength physical performance [10]. Muscle ultrasound is a simple method to evaluate muscle mass in a consultation or at the bedside in hospitalized patients; it is an economic and non-invasive test and allows us to assess several muscular groups. These new approaches, including other techniques such as bioelectrical impedance analysis (BIA), dynamometry, or functional tests (for example, chair test, time up, and go test) to measure functionality could be included in usual clinical practice [7] in order to realize a holistic evaluation of the patient. It is also interesting to evaluate patients with structured nutritional tests that combine different parameters, such as the Mini Nutritional Assessment Short Form (MNA-SF) and the Subjective Global Assessment (SGA). The SGA and MNA-SF are considered adequate tools to diagnose malnutrition, with predictive value for mortality [11]. Finally, new biomarkers can help us in this morphofunctional assessment. For example, serum resistin levels [12] are associated with low skeletal muscle mass in obese women over 60 years of age and other potential molecules need attention in this area [13].

To summarize, it is necessary to implement this new concept of nutritional evaluation in the management of patients and in clinical research in nutrition. Thus, the implementation of these tools is recommended to improve diagnosis, treatments, and patient outcomes in the field of DRM [14].
